# Estimating the Size of Dog Populations in Tanzania to Inform Rabies Control

**DOI:** 10.3390/vetsci5030077

**Published:** 2018-09-07

**Authors:** Maganga Sambo, Katie Hampson, Joel Changalucha, Sarah Cleaveland, Tiziana Lembo, Kennedy Lushasi, Eberhard Mbunda, Zacharia Mtema, Lwitiko Sikana, Paul C.D. Johnson

**Affiliations:** 1 Ifakara Health Institute, P.O. Box 53, Ifakara, Tanzania; jchangalucha@ihi.or.tz (J.C.); klushasi@ihi.or.tz (K.L.); zmtema@ihi.or.tz (Z.M.); lwsikana@ihi.or.tz (L.S.); 2 Boyd Orr Centre for Population and Ecosystem Health, Institute of Biodiversity, Animal Health and Comparative Medicine, University of Glasgow, Glasgow G12 8QQ, UK; Katie.Hampson@glasgow.ac.uk (K.H.); Sarah.Cleaveland@glasgow.ac.uk (S.C.); Tiziana.Lembo@glasgow.ac.uk (T.L.); Paul.Johnson@glasgow.ac.uk (P.C.D.J.); 3 Nelson Mandela African Institution of Science and Technology, P.O. Box 447, Arusha, Tanzania; 4 Department of Epidemiology, Ministry of Livestock and Fisheries, P.O. Box 2870, Dodoma, Tanzania

**Keywords:** dog-mediated rabies, transects, dog ownership, mass dog vaccination

## Abstract

Estimates of dog population sizes are a prerequisite for delivering effective canine rabies control. However, dog population sizes are generally unknown in most rabies-endemic areas. Several approaches have been used to estimate dog populations but without rigorous evaluation. We compare post-vaccination transects, household surveys, and school-based surveys to determine which most precisely estimates dog population sizes. These methods were implemented across 28 districts in southeast Tanzania, in conjunction with mass dog vaccinations, covering a range of settings, livelihoods, and religious backgrounds. Transects were the most precise method, revealing highly variable patterns of dog ownership, with human/dog ratios ranging from 12.4:1 to 181.3:1 across districts. Both household and school-based surveys generated imprecise and, sometimes, inaccurate estimates, due to small sample sizes in relation to the heterogeneity in patterns of dog ownership. Transect data were subsequently used to develop a predictive model for estimating dog populations in districts lacking transect data. We predicted a dog population of 2,316,000 (95% CI 1,573,000–3,122,000) in Tanzania and an average human/dog ratio of 20.7:1. Our modelling approach has the potential to be applied to predicting dog population sizes in other areas where mass dog vaccinations are planned, given census and livelihood data. Furthermore, we recommend post-vaccination transects as a rapid and effective method to refine dog population estimates across large geographic areas and to guide dog vaccination programmes in settings with mostly free roaming dog populations.

## 1. Introduction

Dog-mediated rabies is a serious zoonosis responsible for at least 59,000 human deaths every year, primarily in low-income countries in Asia and Africa where rabies is endemic [1]. In these areas, over 95% of human rabies deaths result from bites by domestic dogs. Empirical data and mathematical modelling have shown that annual dog vaccination campaigns that achieve a coverage of 70% are sufficient to eliminate rabies [2,3]. The global call for the elimination of dog-mediated human rabies by 2030 [4], has prompted many countries to invest in dog vaccinations. For example, Tanzania has developed a National Rabies Control and Elimination Strategy, aiming to control dog rabies and eliminate human rabies in the country by 2030 [5]. However, in low-income countries, dog population sizes are usually unknown, or hard to estimate, making it difficult to implement and evaluate dog vaccination campaigns [6].

In most low-income countries where rabies is endemic, owned dogs are not registered by local authorities, dogs are free to roam, and dog censuses are not conducted. Insufficient knowledge of dog population sizes for planning of vaccination campaigns was reported as one of the limiting factors for ineffective rabies control in Africa [6]. This lack of knowledge prevents countries from forecasting vaccine procurement needs, and hinders assessment of the effectiveness of mass dog vaccination campaigns. It is, therefore, important to develop practical methods for estimating dog population sizes.

Approaches for estimating dog population sizes include extrapolation from human/dog ratios derived from household surveys and from transects with counts of dogs differentiating unvaccinated and vaccinated dogs marked with collars or paint sprays [7,8,9,10,11,12]. The accuracy of these methods has been questioned, as they generate very different population estimates from the same geographical areas, and surveys can be imprecise unless large numbers of households are sampled [7,13]. Household surveys are restricted to assessing owned dogs, while transects capture only free-roaming (observable) dogs. In Tanzania, over 78% of dog owners reported that their dogs roam freely all the time [14], and therefore, the majority of dogs can be observed during transects. In settings where the vast majority of dogs are owned, such as Tanzania [6,14], a complete dog census, whereby each household in a community is visited, is the gold standard method to estimate the dog population, but requires considerable investment of time and resources.

Dog ownership patterns are not uniform across all communities. The distribution of dogs in different settings is linked to religious, cultural, geographical, and socioeconomic factors [9,10,15]. For example, there tend to be fewer dogs in predominantly Muslim communities than in Christian communities [9,15]. Tools to accurately estimate dog populations that take into account local cultural norms could, therefore, support the scaling up of mass dog vaccination programmes.

Our overall aim is to provide practical and effective approaches to estimate dog populations in different settings. Our first objective was to compare methods to determine which provides the most precise dog population estimates, and explain why estimates differ according to method. From this comparison, we identified that post-vaccination transects, which involve counting both vaccinated and unvaccinated dogs, provide more reliable dog population estimates than either household or school-based surveys. Our second objective was to identify factors that predict dog ownership in different settings in Tanzania, using our estimates of dog population size from transects. The aim of identifying these factors was to enable prediction of dog population sizes and densities in other parts of the country not yet subject to vaccination campaigns, which was our third objective. We assessed the performance of these factors from known populations in our study, and finally used these factors together with nationally available human census data to predict dog population sizes throughout Tanzania. Our findings should be valuable for both Tanzania and other countries as they develop, implement, and monitor their national rabies control programmes.

## 2. Methods

### 2.1. Study Sites

**Figure 1:**
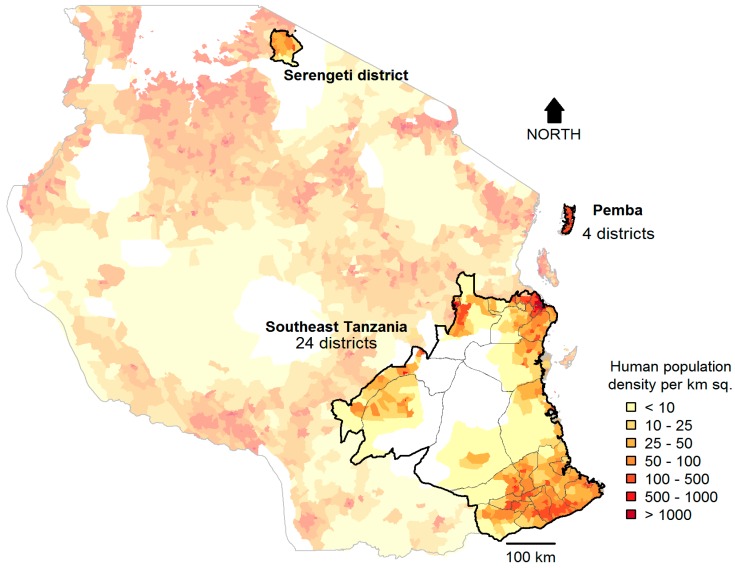
Districts in Tanzania where post-vaccination transects, household, and school-based surveys were conducted. Data were collected in Southeast Tanzania and Pemba (28 districts) where mass dog vaccination campaigns have been conducted since 2011. In Serengeti district, mass dog vaccination campaigns and transects were also conducted in the last quarter of 2015, to assess the performance of the model at predicting dog numbers outside the study area. White coloured areas represent uninhabited protected areas.

**Figure A1:**
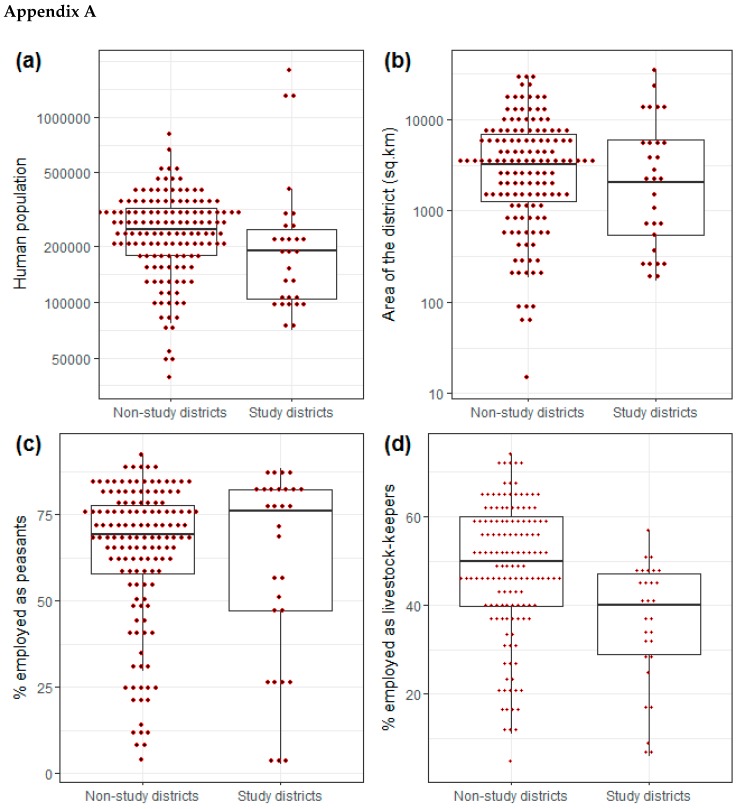
The distribution of the non-study districts and study districts in Tanzania. Red points represent data from each district. (**a**) Human population from the National census in 2012; (**b**) geographical area of the district in square kilometres; (**c**) percentage of population (aged >10 years and above) employed as peasants; and (**d**) percentage of population (aged >10 years and above) employed as livestock keepers.

The study was conducted in 28 districts across Tanzania: 24 from southeast Tanzania and four from Pemba Island (Figure 1). In 2012, the national human population and housing census recorded 8,819,886 people (average population growth rate of 2.16% per year) living in 2,159,721 households in these districts, with 784,424 of these households in rural areas [16]. These districts span a wide range of settings, livelihoods, and religious backgrounds [5], and cover rural, urban, coastal, and inland areas. The districts cover a total area of 165,043 km^2^ (17% of the landmass in Tanzania) and are representative of much of Tanzania in terms of population sizes, area, and livelihoods (Figure A1).

### 2.2. Data Collection

Since 2011, dog rabies vaccinations have been conducted in villages across the study districts using a central point approach [17], whereby owners bring their dogs to a centrally located vaccination point within their village. At the vaccination points, all dogs that were vaccinated were fitted with temporary plastic collars around their necks. We used the number of vaccinated dogs in each district as the minimum possible dog population size from which to compare the performance of our estimates. Data from three different approaches were used to estimate dog populations: post-vaccination transects, household surveys, and school-based surveys. These methods are described in full elsewhere [13], and outlined briefly below.

*Post-vaccination Transects:* Since 2013, following vaccination campaigns, post-vaccination transects have been conducted [13]. Transects were walked (or occasionally cycled) on the same day as campaigns from 16:00 to 18:00, when dogs were active and visible, counting all marked (vaccinated) and unmarked (unvaccinated) dogs. In rural areas, transects were conducted in two randomly selected subvillages per village (villages ranged in size from 2 to 10 subvillages, with a median of 4), aiming to representatively sample each village. In the first subvillage, enumerators started at the subvillage centre and headed to the outskirts, while in the other, enumerators walked from the edge toward the centre. Each transect was conducted by one enumerator for 1 h per subvillage, taking 2 h to complete the village. In urban areas, enumerators covered the jurisdiction of a street (a geographical area defined by the National Census, which covers a neighbourhood with several roads). Enumerators selected the direction at the start of transects, at the border of subvillages/streets and at road junctions by spinning a pen. Numbers of vaccinated dogs per district were compiled from dog vaccination registers and used in conjunction with the transect data to estimate coverage achieved and numbers of dogs in each district. In Serengeti district in northern Tanzania, data from mass dog vaccinations and transects that were conducted in August 2015 were used to validate the performance of transects. Data from a complete census of dogs (covering all 35,867 households in Serengeti district), undertaken from 2008 to 2015, were used to calculate the pup/adult ratio of dogs.

*Household Surveys:* Household surveys were completed in all 28 study districts between July and August 2011, with the aim of obtaining an initial assessment of coverage from the first phase of vaccination campaigns conducted between February and April in 2011. Six villages were randomly selected from all villages in each district. In each selected village, a landmark was identified (preferably a school, otherwise a dispensary, church, or mosque). From this starting point, interviewers randomly chose a direction for selecting households for interview by spinning a pen. Every third household was sampled, and interviews conducted until 30 households were completed in each village. For households that owned dogs, the questionnaire captured details of dogs owned (adults and puppies <3 months) and their vaccination status on the basis of owner recall.

*School-Based Surveys:* School-based surveys were conducted from June 2014 to February 2015, in the two months following dog vaccination campaigns in each district. The surveys were conducted in 24 districts (4 districts were missed) and were used to ask questions on the number of adults and children (<18 years of age) in households, as well as the number of dogs and puppies (<3 months of age), and their vaccination status. Six primary schools (one per village; most villages in Tanzania have a primary school) were randomly selected from each district but logistic and financial limitations meant that surveys were not conducted in four districts and were conducted in fewer than six schools per district as initially planned. We used total population purposive sampling with a target to interview 100 pupils per school [18]. Standard VII pupils (ages from 13–15 years) were selected to complete the questionnaire. If more than one pupil from a household was recruited, the oldest pupil was selected. If the school had fewer than 100 standard VII pupils, pupils were recruited from lower classes (Standard IV–VI, ages from 12–14 years). Schools were selected from the same villages where household surveys were conducted.

### 2.3. Characteristics of Study Districts

Human demographic data for the study were extracted from the 2012 national population and housing census, including district-level population sizes, annual population growth rates, average household sizes (persons/household), numbers of livestock keeping households, and the percentage of the population living in rural areas [16]. Human populations were projected from the 2012 census to 2014, using annual growth rates for each district. The census also included district-level information on employment status among the working population (defined as those aged 10 years or over), which showed that the most frequent occupations were farming and livestock keeping. We, therefore, also extracted the number of workers in each district employed as peasants and livestock keepers, respectively. To examine whether geographical setting was associated with dog ownership, districts were also categorized as (1) *Inland*, comprising 14 districts from the mainland that did not border the ocean; (2) *Coastal*, comprising 14 districts that bordered the Indian Ocean, including both Tanzania mainland coastal districts and the four districts from Pemba island; and (3) *Island*, covering the four districts from Pemba island. We extracted the geographical area of each district from data obtained from the National Bureau of Statistics [19], using the *unionSpatialPolygon* function from the *maptools* package in R [20], excluding water bodies and protected areas. These variables are summarised in Table 1 for study and non-study districts.

### 2.4. Statistical Analysis

*Estimation of dog population sizes:* For transects, which involve counting both vaccinated and unvaccinated dogs, estimates of dog vaccination coverage, and their 95% confidence intervals (CIs), were obtained from binomial generalized linear mixed models (GLMMs) with normally distributed (on the logit scale) random intercepts allowing coverage to vary among villages. Dog population estimates for each district were calculated by dividing the numbers of vaccinated dogs by the coverage estimates. Puppies under the age of three months are not often vaccinated during campaigns [21,22], and are less likely to be counted than adult dogs during transects [13,22,23]. In Thailand, puppies comprised approximately one quarter of the dog population [10]. Estimates of population sizes from transects therefore require further adjustment. We calculated the ratio of pups/adult dogs (1:3.81) from the dog census conducted in Serengeti district in northern Tanzania [13], and adjusted our population size estimates accordingly. We used transect and mass dog vaccination data collected between November 2014 to January 2015 for these calculations. Transect data were collected from 27 districts (we were unable to collect transect data in Ilala district, and it was not included in our analysis). To assess year-to-year variation of the estimated dog population sizes, we compared these data (November 2014 to January 2015) with data that were collected from September 2015 to December 2016. 

The total number of dogs across the 27 districts was calculated as the sum of the dog population estimates in each district. A 95% confidence interval for this total was estimated as the 2.5% and 97.5% quantiles from 100,000 bootstrap samples, where bootstrapped samples for each district were generated by first sampling coverage estimates from a normal distribution with the mean being the estimated log odds of coverage and the standard deviation being its estimated standard error, and second, converting these coverage estimates to total dog population sizes, with adjustment for undercounting of puppies, as described above.

For household and school-based surveys, we estimated dog population sizes by multiplying the mean number of dogs per household recorded during these surveys by the total number of households within each district according to the 2012 national census. For each district, the mean number of dogs per household and profile likelihood 95% confidence limits were estimated from a negative binomial GLMM, where the response was the number of dogs in each household, and the only fixed parameter was the intercept. Confidence limits were not calculated for districts where too few (≤3) surveyed households owned dogs to provide reliable estimates of uncertainty. We explored fitting a random effect to account for variation in dog numbers among villages, but omitted it because, for the majority of districts, the model either failed to converge, suggesting insufficient information in the data to estimate the variance, or gave zero variance estimates and, therefore, identical estimates and confidence intervals to the model, with no random effects. The district-level estimates from the household and school-based surveys were summed to estimate the overall dog population size in the study districts. Confidence intervals for these estimated totals were calculated by parametric bootstrapping, similarly to the transect methods above, by sampling from a normal distribution with the mean being the log mean number of dogs per household, and the standard deviation being its standard error estimated from the GLMM. Where too few households owned dogs to allow the GLMM to be fitted, the standard error was assumed to be zero. 

*Prediction of dog population sizes:* We used a linear regression model to achieve our second objective of identifying factors (those considered are shown in Table 1) associated with dog population sizes, and our third objective of estimating the number of dogs in each district. Here, we motivate our approach to modelling dog numbers. 

First, rather than modelling dog numbers directly, we modelled the dog/human ratio, then multiplied the estimated dog/human ratio by human population size to estimate dog numbers. A simple model for predicting the ratio of the number of dogs (*D_i_*) to the number of humans (*P_i_*) in district *i* is $\frac{D_{i}}{P_{i}}=K\varepsilon_{i}$, where every district is predicted to have the same dog/human ratio, *K*, from which individual districts are allowed to deviate by means of district-specific residuals, *ε_i_*, where *ε_i_* > 0 with geometric mean 1. We can add flexibility to this model by introducing continuous variables that are potential predictors of the dog/human ratio, for example, the proportion of the population employed as livestock keepers, *L_i_/P_i_*, such that $\frac{D_{i}}{P_{i}}=K{\left(\frac{L_{i}}{P_{i}}\right)}^{\beta_{1}}\varepsilon_{i}$, where *β*_1_ is a coefficient governing the influence of *L_i_/P_i_* on *D_i_/P_i_*. The model can be further extended to allow differences in *D_i_/P_i_* between categories of district, such as between districts with and without a coastline: $\frac{D_{i}}{P_{i}}=K{\left(\frac{L_{i}}{P_{i}}\right)}^{\beta_{1}}\exp\left(\beta_{2}C_{i}\right)\varepsilon_{i}$, where *C_i_* is a binary indicator variable with the value 1 for coastal districts and 0 otherwise, so that exp(*β*_2_) represents the ratio (coastal dog/human ratio)/(mainland dog/human ratio). Taking the natural log shows that this model can be viewed as a linear regression model: $\log\left(\frac{D_{i}}{P_{i}}\right)=\log\left(K\right)+\beta_{1}\log\left(\frac{L_{i}}{P_{i}}\right)+\beta_{2}C_{i}+\log\left(\varepsilon_{i}\right)$, where log(*ε_i_*) ~ Normal(0, *σ*^2^). Further continuous and binary variables (listed in Table A1) were added in the same way (continuous variables were logged, and categorical variables fitted as binary indicator variables) to give the full model prior to filtering using the variance inflation factor.

We used this ordinary least squares regression model to assess whether any of the variables that we collated were useful for predicting numbers of dogs. We partitioned our data into two sets of districts: a model-building set, which we used to develop the model, and a prediction set, for which we aimed to predict dog numbers from the model. The model-building set comprised the data from the 28 study districts where we collected data, whereas the prediction set included all districts in Tanzania (including the 28 study districts). The response variable was the estimated number of dogs in each of the study districts, log-transformed to fit the modelling assumptions of linearity and homoscedasticity. Before fitting the model, we conducted data exploration to detect collinearity among the variables using pairwise scatter plots and the variance inflation factor (VIF) as described by Zuur et al. [24]. The variable with the highest VIF was removed and VIFs recalculated, repeating this process, stopping when all VIF values were below 5. The remaining variables were taken forward to model selection.

The aim of model selection was to identify the best-fitting model, that is, the model that most accurately predicted district dog population sizes. The best model was selected by fitting all possible subsets of the six candidate variables as main effects, and choosing the model with the lowest corrected AIC (AIC_C_) [25]. The predictive power of the best model was assessed by calculating *R*^2^_FPE_, where FPE stands for final prediction error [26]. The interpretation of *R*^2^_FPE_ is similar to classical *R*^2^ and adjusted *R*^2^, except that unlike these two statistics, *R*^2^_FPE_ does not overestimate predictive power [26]. We also calculated adjusted *R*^2^ for comparison. The contribution of each selected variable to the predictive power of the best model was gauged by calculating partial *R*^2^_FPE_, which estimates the proportional reduction in prediction error when a given variable is included in the final model. Partial *R*^2^_FPE_ is calculated as 1 − (1 − *R*^2^_FPE_)/(1 − *R*^2^_FPE_*), where *R*^2^_FPE_ is calculated from the best model, and *R*^2^_FPE_* is calculated from a reduced version of the best model, where the variable under investigation has been dropped.

Two methods were used to assess the validity of the final model. First, we assessed the propensity for the model selection procedure to select spurious variables (“false discoveries”). We permuted the response variable 1000 times to simulate 1000 datasets in which none of the variables are associated with the response. We applied the model selection procedure to each dataset and calculated the mean number of variables selected. The ratio of mean number of false discoveries to the actual number of variables selected is a permutation-based estimate of the false discovery rate (FDR; [27]). Second, we used bootstrapping to assess the stability of the selected variables to sampling error. We performed the model selection procedure on 1000 bootstrapped datasets (datasets of equivalent size but sampled with replacement from the original dataset), recording the proportion of datasets from which each variable was selected. The final model was then used to predict the dog population size, density, and 95% prediction interval for each district in Tanzania.

To assess year-to-year variation in our estimates, we compared dog population sizes estimated from data that were collected in 2014–2015 against those estimated from 2015–2016 data. 

When making predictions from the models of coverage and the number of dogs per 1000 humans, it was necessary to back-transform from the linear predictor scale using a nonlinear inverse “link” function (inverse logit for coverage and exponential for dog numbers). Since these models have normally distributed errors, predictions would be biased, potentially severely, if calculated simply by back-transforming without adjusting for the error variance, as a consequence of Jensen’s inequality [28]. Log-scale predictions from the model of district dog population size were adjusted for Jensen’s inequality by back-transforming using exp(μ + 0.5σ^2^), and logit-scale predictions of vaccination coverage were back-transformed using logit^−1^(μ + 0.5σ^2^ tanh(μ(1 + 2 exp(−0.5σ^2^))/6)), where μ is the link-scale prediction and σ^2^ is the total error variance [29].

All statistical analyses were conducted using R version 3.4.1 [30]. GLMMs were fitted using the lme4 [31] and *glmmTMB* packages [32]. Models selection was performed using the *dredge* function from the *MuMIn* package [33].

### 2.5. Ethics

The study protocol was approved by the Medical Research Coordinating Committee of the National Institute for Medical Research of Tanzania (NIMR/HQ/R.8a/Vol.IX/2109), the Institutional Review Board of the Ifakara Health Institute, and the Tanzania Commission for Science and Technology (COSTECH). Before administering any questionnaires, participants were informed about the background and purpose of the study. Participants were informed that they were free to participate in the study and withdraw from the study at any time during the interview, and that their answers would be kept confidential.

## 3. Results

During the 2014–2015 mass dog vaccination campaigns, 86,361 dogs were vaccinated in the 28 study districts, and 86,142 dogs were vaccinated in the 2015–2016 campaign. The following data collection activities were completed: (i) post-vaccination transects in ~2100 villages in 2014–2015 and in ~2600 villages in 2015–2016; (ii) household surveys in 4488 households in 2011, from 160 randomly selected villages; and (iii) school-based surveys of 8254 primary school pupils (each representing a unique household) within 115 randomly selected schools following the 2014–2015 campaign. During the 2014–2015 transects, 18,436 dogs were counted, of which 63% were observed with collars, indicating that they were vaccinated. From the school-based surveys, 2198 owned dogs were reported, corresponding to a mean of 0.7 dogs per household, and from the household surveys, 731 dogs were recorded, corresponding to a mean of 0.6 dogs per household (Table A2).

**Figure 2:**
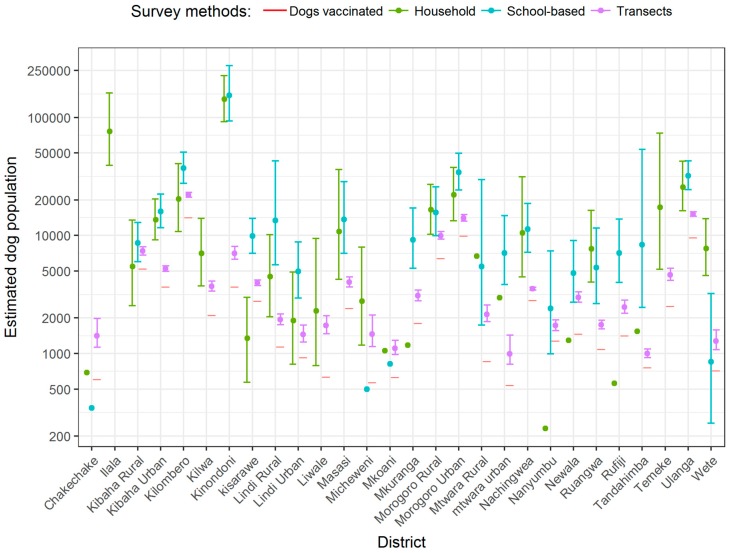
Comparison of the estimated number of dogs in the 28 districts from the three methods: household surveys, school-based surveys, and transect surveys. Estimates are shown on a log scale (the actual scale is shown in Figure A3). Error bars show 95% confidence intervals (CI) around the mean estimate. Household surveys were conducted in 2011, while school-based surveys and transects were conducted in 2014 and 2015 following dog vaccination campaigns. The number of vaccinated dogs from the 2014–2015 campaign is also plotted as a horizontal line for each district. CIs are not plotted for household or school-based surveys from districts, where fewer than three households were recorded to own dogs, among the randomly selected households surveyed in each district.

**Figure A2:**
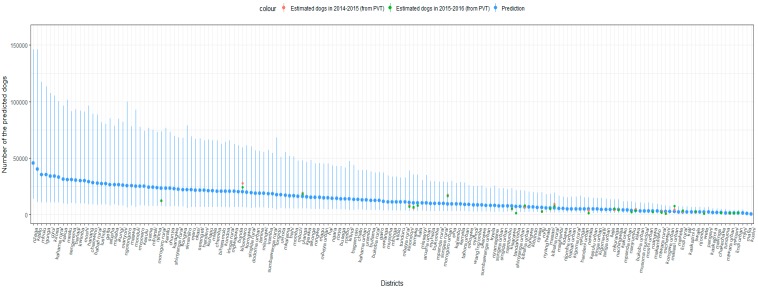
The predicted number of dogs in districts across Tanzania. Model predictions (points) and their prediction interval are shown in blue and compared to dog populations estimated directly from transects conducted in 2014–2015 (red points) and in 2015–2016 (green points). This comparison shows that there was minimal year-to-year variation in the estimated number of dogs per district.

*Estimation of dog population sizes:* The overall dog population estimated in these study districts varied according to the survey method used (Figure 2). From transects, we estimated a total dog population in the 27 study districts of 164,000 (95% CI 163,000–169,000 reported to three significant digits) and an overall human/dog ratio of 53.6:1 in the study districts. The estimated dog population size leads to a vaccination coverage estimate of 52% (86,000/164,000), which is lower than the direct coverage estimate from transects (63%), due to adjusting for unobserved and unvaccinated pups. By contrast, using household and school-based surveys, we estimated the dog population in the study districts to be 412,000 (CI 348,000–544,000) and 403,000 (CI 341,000–531,000), respectively. District-level estimates of dog numbers from household and school-based surveys tended to have wide 95% confidence intervals, with a mean ratio of upper to lower confidence limit of 3.5 for household, and 3.0 for school-based surveys. Transect estimates were considerably more precise, with a mean upper/lower confidence limit ratio of 1.2. Household and school-based surveys were also sometimes highly inaccurate, giving estimates that were lower than the number of vaccinated dogs (Figure 2). Transect estimates, by contrast, cannot be lower than the number of vaccinated dogs. We therefore considered transect estimates to be more reliable, and used them to fit the model for predicting dog population sizes. There was minimal year-to-year variation in the estimated number of dogs in each district from the data that were collected in 2014–2015 versus those collected in 2015–2016 (Figure A2).

*Prediction of dog population sizes and densities:* using our transect estimates, we investigated the influence of district-level variables on dog population sizes. The pairwise plots between the log-scale continuous variables investigated showed that the number of households was highly correlated with the human population (Pearson’s *r* = 0.96). We therefore dropped the number of households from the model. A further two variables, the number of people living in rural areas and the number of livestock-owning households, were dropped in order to reduce all VIFs to below 5 (Table A1). These variables were also highly correlated with human population size.

All 64 possible models were fitted from the combination of the six retained variables and the models were ranked by δAIC_C_ (Table 2). The top three models were almost equivalent in predictive power (*R*^2^_FPE_ = 58%), and were very close in δAIC_C_, which ranged from 0–1.27. Our best fitting model retained three variables: the proportions of livestock keepers and of peasants, and the geographic setting (inland versus coastal and island). The proportions of livestock keepers and peasants were both positively associated with the dog/human ratio: a doubling of the proportion of livestock keepers was associated with 28% (95% CI: 14%, 44%) larger dog populations, while the equivalent effect for the proportion of peasants was 36% (95% CI: 13%, 65%), all other characteristics being equal. We also found that there were 103% (95% CI: 21%, 120%) more dogs per person in inland districts than in island and coastal districts. 

Two predictor variables: the proportion of livestock keepers and the geographic setting (inland versus coastal/island) were consistently retained in the best-ranked models (Table 2). Dropping either the setting variable or the proportion of persons employed as peasants also reduced the variance explained by 16% (*R*^2^_FPE_ fell from 58% to 42% in both cases, giving a partial *R*^2^_FPE_ of 27%). Excluding the proportion of persons employed as livestock keepers in the final model reduced *R*^2^_FPE_ to 30%, showing the substantial predictive power of this variable (partial *R*^2^_FPE_ = 39%). 

We used permutations and bootstrapping to assess the reliability of the selected best-fitting model. Using 1000 permutations, we estimated an FDR of 28%. Bootstrapping the model selection procedure showed that two of the three variables were highly robust: proportion of livestock keepers and coastal setting were selected in 97% and 91% of bootstrapped models, respectively (Table 2). However, despite their robustness, a model containing these two predictor variables alone performed substantially worse than the best-fitting model, as evidenced by its relatively low *R*^2^_FPE_ of 42% and its selection as the best model in only 6% of bootstrapped datasets. Two other variables were selected with around 50% frequency, proportion of the population employed as peasants (49%), and human population size (51%). Based on δAIC_C_, *R*^2^_FPE_, and the reliability analysis, we concluded that two of the three variables selected are highly robust, and that at least one other variable is required to maximise predictive power. Since the top three models are almost equivalent in terms of predictive power (*R*^2^_FPE_) and δAIC_C_, we selected the one with only three variables selected, which also happened to be the best-fitting model. This combination of three variables (the proportions of livestock keepers and of peasants, and geographic setting) chosen for our final model was used to predict the dog population across Tanzania.

**Figure 3:**
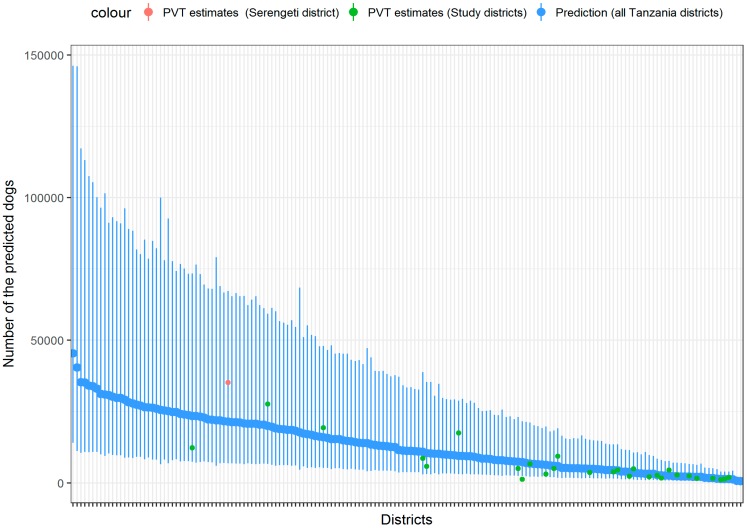
The predicted number of dogs in districts across Tanzania. The predictions and prediction intervals are shown in blue and compared to the dog population estimated directly from transects for a non-study district (Serengeti, in red) and for the study districts. Most of the green points (27 out of 28) from the model-building set were within the prediction intervals, suggesting that the fit of the model is reasonably good, with no outlier districts for whom the model is making poor predictions. Districts are ordered according to those with the largest (left-hand side) to the smallest predicted dog population (right-hand side). District names are shown in Figure A2. Serengeti transects were conducted in August 2015. PVT = post-vaccination transects.

From our final model, we predicted considerable variability in dog population sizes across all 168 districts in Tanzania (Figure 3). Dog population estimates ranged from just 630 dogs in Kusini district on the island of Zanzibar, to 45,000 dogs in the inland district of Nzega. Overall, the final model predicted a dog population of 2,316,000 (95% CI 1,573,000–3,122,000) in Tanzania in 2014–2015. In 2014, a human population of 47,831,000 was projected from the Tanzania population census [16]. Taken together with our predicted dog population size (2,316,000 dogs), we obtain an overall human/dog ratio of 20.7:1 in Tanzania. Generally, the highest dog ownership was predicted from inland districts, especially those dominated by rural livestock keepers. Human/dog ratios for each study district are presented in Table A2.

**Figure 4:**
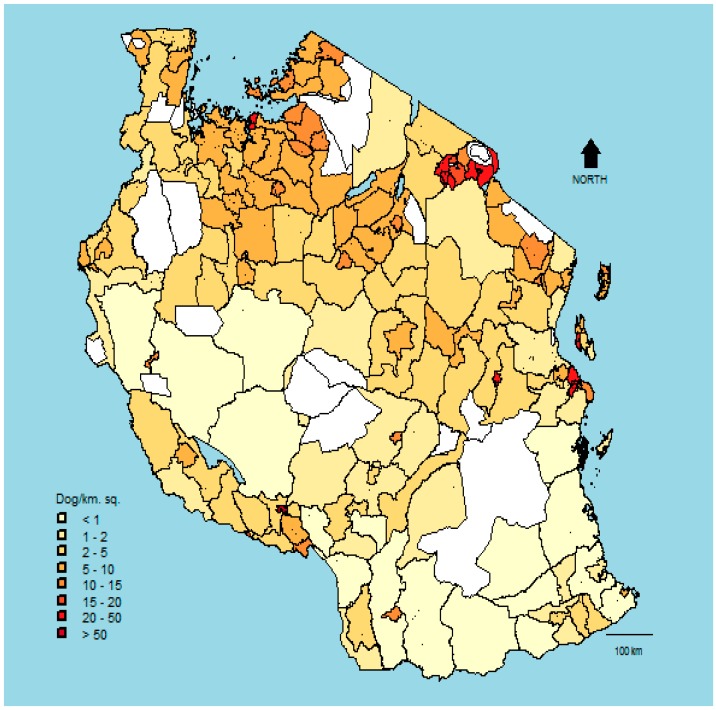
Estimated dog densities in districts across Tanzania. White areas represent water bodies, forest reserves, or wildlife-protected areas.

Dog density from the predicted dog population sizes for each of the 168 districts also varied greatly, ranging from 0.14 to 113 dogs per square kilometre (Figure 4). Liwale district (an inland study district which has an area of ~15,000 km^2^) had the lowest dog density of 0.14 per km^2^, while Bukoba urban (an inland district with an area of 30 km^2^) had the highest dog density of 113 dogs per km^2^. Based on Tanzania’s total land area and predicted dog population, we determined the mean density of dogs to be 8.81 per square kilometre (interquartile range: 2.24–9.23 per km^2^). Predicted densities were highest in northern and northeastern Tanzania, and lowest in the southern and central-west parts of the country (Figure 4). 

## 4. Discussion

Knowledge of the size of dog populations is critical for the planning and implementation of effective canine rabies control strategies. Before implementation of vaccination campaigns, this information is useful for determining the personnel required, and for the procurement of vaccines and other supplies. After implementation, this information is required to evaluate the intervention in terms of the vaccination coverage achieved. Our study uses data from well-studied dog populations in Tanzania, where vaccination campaigns have been conducted to predict the size of dog populations in new areas, and where vaccination campaigns will hopefully be scaled up in the future.

Although several methods have been used to calculate the size of dog populations [34], these methods have not been comprehensively compared to assess which generates the most precise dog population estimates. From our comparison, we found that post-vaccination transects generated more precise and reliable estimates than either household or school-based surveys in Tanzania. Transects are, however, only reliable if all dogs (owned and unowned, free roaming or restricted) have an equal chance of being counted. In many sub-Saharan countries, most owned dogs are free roaming, so this assumption would hold. In a previous survey in Tanzania, most dog owners reported that they did not tie or cage their dogs. Those who reported restricting their dogs were from urban areas, while in rural areas, the vast majority of dog owners reported that they do not restrict their dogs at any time [14]. Hence, transects are appropriate for estimating dog population sizes in Tanzania, but in countries where a large proportion of dogs are kept indoors, this method would not be appropriate.

The imprecision of the dog population estimates from household and school-based surveys was due to their low sampling effort compared with post-vaccination transects [13]. Although sample size at the household level was not low (30 households per village and ~100 families per school), only six out of an average of 95 villages per district were sampled, and the precision of estimates from hierarchical sampling designs is expected to be dominated by sample size at the highest level [35,36]. The overall proportions of the population surveyed were also small (4488/441,000 households from household surveys versus 8254/441,000 households from school-based surveys), despite the large numbers of surveys conducted, whereas for the post-vaccination transects, ~2500 villages were sampled. The practical consequence of imprecision in the estimates from household and school-based surveys for the implementation of mass dog vaccination is that the upper bound of the range of plausible dog population sizes is, on average, 3 times the lower bound, compared with 1.18 for transects.

In addition to being imprecise, estimates from household and school-based surveys were often inconsistent (Figure 2), either with each other (for example, Kisarawe and Wete districts), or with the number of dogs vaccinated (for example, Mkuranga and Chakechake districts). The dog population may, therefore, be either over- or underestimated by projecting from the mean number of dogs per household, given limited sampling and considerable house-to-house variation in dog ownership. For example, Kinondoni district in Dar es Salaam has over 400,000 households with 0.32 dogs per household estimated from the household survey, which would suggest a dog population of 141,197, a large overestimate (Table A2).

The number of vaccinated dogs (numerators), together with the size of dog populations derived from household and school-based surveys (denominators), were used to calculate the vaccination coverage, and compared against coverage estimated directly from these surveys. We found large discrepancies depending upon the method used. For example, in 2014–2015, around 86,000 dogs were vaccinated, but school-based surveys estimated a dog population of 399,000, which would have led to just 22% coverage (Table A2). By contrast, direct estimates of dog vaccination coverage from school-based and household surveys were both around 56% (Table A2). In some districts, the discrepancy was clearly implausible, for example, in Chake Chake, Pemba, where more than double the number of dogs estimated from school-based surveys were vaccinated (Table A2). These discrepancies have practical impacts on rabies control, and demonstrate the need for careful post-vaccination evaluations, without reliance on dog population survey estimates only. 

Our multivariable regression analysis identified the proportion of livestock keepers and geographical setting as robust variables for predicting dog population sizes, consistent with previous studies on factors influencing dog ownership in different parts of the world [34,37,38]. In most African countries, dogs are reported to play a role in protecting livestock, explaining why livestock keeping was such an important variable [9,15]. Other factors reported to influence dog ownership in Africa include socioeconomic status, livelihood (which could include livestock keeping), culture, and religious beliefs linked to different settings. The reason for fewer dogs in Tanzanian coastal and island areas could be linked to the predominantly Muslim communities in these areas, as Muslims are reported to own fewer dogs [9,15]. Our study demonstrates the need to consider such factors in planning vaccination campaigns, and in understanding dog rabies incidence, control, and prevention, more generally.

We found that despite the robustness of these two variables (livestock keeping and geographical setting), by themselves, they accounted for only about two-thirds of the predictive power of the best-fitting model. A third variable was also needed to improve predictive power. Our model validation showed that the proportion of peasants or the human population were almost equivalent in the final model (*R*^2^_FPE_ values of 58% and 55%, respectively). If applying this model to new settings, the decision as to which variable to use will depend on the availability of data on either of these variables. We also do not propose evaluating campaign success by working backwards from the total dog population estimates derived from this predictive model, because the uncertainty in this estimate is inflated from the large variation in district population sizes. To evaluate coverage across districts, we would suggest estimating mean coverage directly from the village-level transect measures. District-level estimates of dog populations (Figure 2), which are based on these coverage estimates, are quite precise, as are directly derived district-level estimates of coverage ([13], 58–65% not adjusted for puppies).

Our study was consistent with previous findings in Tanzania, which reported more dogs in mainland compared to island and coastal areas [9,13]. However, our overall human/dog ratio estimate of 20.7:1 was higher than previous studies in Africa, which ranged from 3:1 to 15:1 [6,9,15,37,39,40,41]. This suggests that human/dog ratios extrapolated from household or school-based surveys could be unreliable when extrapolated to district or national level. Initially, the study area was estimated to have about 400,000 dogs [5,42], based on reported human/dog ratios [9], which was much higher than the number of dogs subsequently estimated with post-vaccination transects (164,000 (95% CI 163,000–169,000). The lower number of dogs in our study suggests that dog vaccine requirements in Africa might be less than previous estimates. A study in Uganda also found lower numbers of dogs than previously estimated [43]. If this pattern holds across more countries, the lower number of dogs provides further incentives for African governments to undertake vaccination programmes, as the target of 70% could be more easily achieved [43]. However, our data were largely collected from southeast Tanzania, where there are fewer pastoralists who tend to own more dogs [44], and from coastal or island districts (~50% of study districts) which tend to have fewer dogs. This suggests that additional data (dog vaccination and transect surveys) from other populations (inside and outside of Tanzania) would be valuable to further refine and validate this predictive approach.

Several household surveys have been conducted in Tanzania, generating lower human/dog ratios than we found from transect-based estimates. For example, in Iringa urban, the human/dog ratios were estimated to be 14 [8], versus our transect estimate for Iringa urban of 34. Meanwhile, in Kilombero and Ulanga districts, human/dog ratios were estimated to be 12 and 29, respectively, from households surveys [15], in contrast to our transect estimates of 21 and 18, respectively. Variation was also reported by geographical setting, with human/dog ratios estimated to on average be 7.6:1 in rural-inland areas, 10.8:1 in rural-coastal areas, 27.1:1 in urban-coastal areas, and 14.4:1 in urban-inland areas [9]. These estimates derived from household surveys are likely to be affected by limited sampling. Mark-recapture studies are useful for estimating numbers of dogs [7]. Our study suggests that transects, when done in association with dog vaccinations at scale, can capture population variability. However, there is still a need for predictive methods for working in areas where dog vaccinations have yet to be conducted, such as the model that we developed. 

Our predictive model could be used to make preliminary predictions of dog numbers in other countries that are similar to Tanzania, with respect to dog-owning practices i.e., where most dogs are free roaming and there are very few unowned dogs. This model can give a starting point for settings with no dog population size estimates even prior to any vaccination campaigns (and transects), given available data on the proportions of livestock keepers and of peasants or on human population sizes. Such preliminary dog population estimates could provide a baseline for planning mass dog vaccinations. The wide confidence intervals of these model estimates may initially mean procurement of excess or insufficient numbers of vaccine vials. However, overprocurement should not be problematic, as the vaccines can be stored for long periods (normally three years) for use in future campaigns. Moreover, subsequently, the vaccination and transect data generated during vaccination campaigns should be used to refine dog population size estimates. Conducting post-vaccination transects in every village is, however, labour-intensive and costly [13], so should not necessarily be undertaken every year. We recommend conducting transects at least in the first years of the undertaking campaigns, to refine dog population estimates. Awareness and participation of dog owners typically increase in the first few years of a rabies control programme [5], so transects may help to refine estimates in the second or third campaigns. However, in subsequent years, established denominators can be used to evaluate the performance of vaccination campaigns as substantial changes in dog population sizes are not expected (Figure A2). We do also recommend repeating transects after several years given dog population growth, and conducting transects in areas where control programmes have been less successful than expected, so that any coverage gaps that may be limiting progress can be identified [13].

Our overall estimates of dog density were higher those reported from elsewhere in Africa [37,45,46,47,48]. This was probably because we excluded water bodies and protected areas when calculating densities. Our dog density map highlights districts with high dog densities that should be prioritized in the scaling up of dog vaccinations (only two districts, Moshi urban and Zanzibar urban, had densities exceeding 120 dogs/km^2^, Table A2), and districts where dog densities might be too low to support rabies transmission without importation from other districts [47,49,50].

Our study had several limitations. The main limitation was that we could not externally validate our predictive model, due to a lack of reliable data on dog numbers outside the study area, with the exception of Serengeti district. In addition, our study areas did not cover many inland livestock keeping districts. Our surveys were also completed at different times, with household surveys conducted in 2011, transects immediately following dog vaccination campaigns in 2014/15, and school-based surveys within 2 months of these dog vaccinations. These differences may have affected our estimates. Transects do also have several limitations. They may result in the recounting of dogs [51], but we tried to avoid this in the design of transect paths. Larger villages also require more time to complete and those with more subvillages were less well sampled. Only observable dogs are counted from transects, which results in systematic biases, such as poor observation of pups [7], but we tried to adjust for this using pup/adult ratios. The pup/adult ratio was calculated from a dog census completed in Serengeti district data between 2008 and 2015. Although this dog census was conducted over multiple years, we do not expect that the dog population structure changed very much during this period. Notwithstanding these limitations, we found that transects were fast and relatively low cost to complete at scale, sampling populations more representatively than other approaches that were limited in spatial scope. We recommend that marking of vaccinated dogs (visible markers/collars) should be included as part of mass dog vaccination campaigns, and that transects should be completed immediately after vaccination campaigns, aiming to cover the centre and the periphery of villages, as coverage has been reported to decrease with the distance to the vaccination point [17,22,52,53]. Estimates should also be adjusted to account for not observing pups.

## 5. Conclusions

Our study underlines the importance of knowledge of dog population sizes and distribution in different settings in rabies endemic countries, with methods of estimating the number of dogs needed to plan, monitor, and evaluate the performance of rabies control efforts. We demonstrated that post-vaccination transects, together with dog vaccination data, can be used to rapidly generate and refine dog population estimates in areas with ongoing vaccination campaigns. Using the transect population estimates, we developed models that use demographic and geographical characteristics to generate tentative predictions of dog populations in districts without dog vaccination interventions. We also show that data derived from smaller scale sampling of the dog population could lead to substantial under- or overestimation of the population in areas with considerable village-to-village variation, leading to poor rabies control. We conclude that post-vaccination transects may be a useful tool for rabies elimination, taking advantage of data on vaccinated dogs that are routinely collected through implementation of mass dog vaccinations.

## Figures and Tables

**Table 1:** Characteristics of study and non-study districts in Tanzania. Continuous variables are summarised by the mean (range) and categorical variables by the number (%). Variables were either extracted from the national census [16] or from district shapefiles from the Tanzania National Bureau of Statistics (NBS) website [19]. These variables were tested using ordinary least squares regression to assess the best variables for predicting numbers of dogs in districts.

Variable	Study Districts (*n* = 28)	Non-Study District (*n* = 140)
Human population size	307,676 (70,209, 1,775,049)	257,188 (39,242, 807,619)
Annual human population growth rate (%)	2.3 (−1.0, 7.0)	2.6 (−3, 7)
Number of households	75,452 (16,892, 441,240)	50,636 (9027, 134,608)
Average household size (persons per household)	4.2 (3.5, 5.5)	5.1 (3.8, 7.8)
Area (km^2^) ^¥^	4375 (15, 28,000)	4375 (18.6, 28,244)
Setting:		
Inland	14 (50%)	128 (91%)
Coastal ^‡^ [Island]	14 (50%) [4 (14%)]	12 (9%) [6 (4.7%)]
Number of livestock-keeping households	18,317 (4771, 35,829)	24,168 (2258, 71,335)
Proportion (%) of persons employed * as:		
Peasants	60 (3, 88)	64 (4, 93)
Livestock keepers	1 (0, 6)	1 (0, 65)

^¥^ Excluding protected areas and water bodies; ^‡^ Coastal districts were defined as districts that border the Indian ocean; * Defined as the main occupation on which persons aged 10 years and above spend most of their working time.

**Table 2:** Regression coefficients and model fit statistics for models predicting dog populations in Tanzania. Statistics are shown for all models with δAIC_C_ < 4, ranked in order of decreasing fit as gauged by δAIC_C_. We report the predictor variable regression coefficient estimates, model fit statistics including the partial *R*^2^_FPE_, the *R*^2^_FPE_^,^, adjusted *R*^2^, the degrees of freedom (df), the log-likelihood (LL), delta AIC (δAIC_C_), and akaike weights. We also report the importance of each predictor variable (variable robustness).

Regression Coefficient Estimates							Model Fit Statistics						
Intercept	Area (km^2^)	Inland vs Coastal & Island	Mainland vs. Island	Number of People	Employed as Livestock Keepers	Employed as Peasants	Partial R^2^_FPE_	R^2^_FPE_	Adjusted R^2^	df	LL	δAIC_C_	Aikakeweight
5.60		−0.708			0.358	0.448	57.6%	61.6%	72.4	5	−18.65	0.00	26.0%
9.51	0.124	−0.758		−0.447	0.303		57.9%	63.1%	76.3	6	−17.49	0.94	16.2%
8.07		−0.760		−0.219	0.335	0.300	57.5%	62.7%	76.0	6	−17.65	1.27	13.8%
9.99		−0.907		−0.412	0.281		55.3%	59.5%	75.7	5	−19.39	1.48	12.4%
11.03		−0.767	−0.422	−0.489	0.302		54.7%	60.2%	70.3	6	−18.54	3.05	5.7%
Variable robustness	33%	91%	13%	51%	97%	49%							

**Figure A3:**
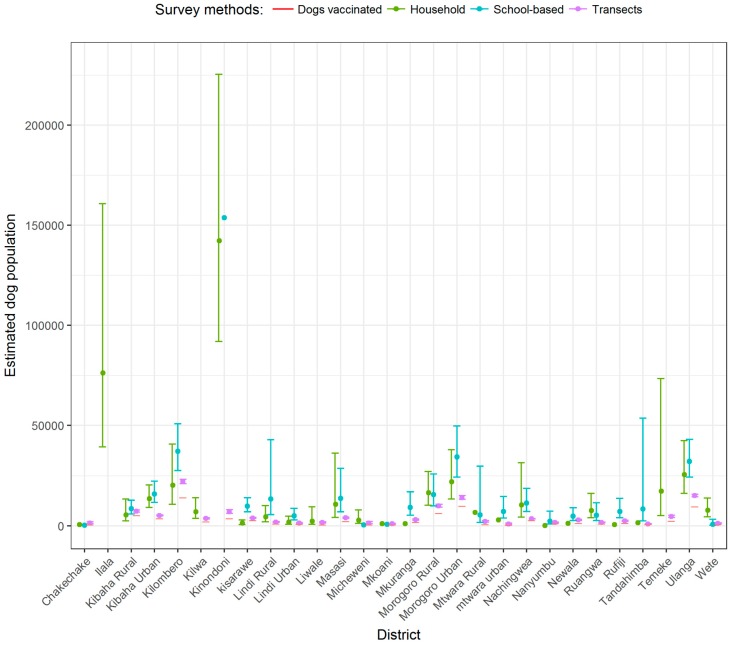
Dog population size estimates for the 28 districts. Data for the household survey were collected in 2011 (different dog vaccination campaigns), while school-based survey and transects were conducted in 2014 and 2015 following the dog vaccination campaigns. Points are the mean estimate and error bars show the 95% confidence intervals (CIs) around the mean. We did not report CIs for districts where less than three dog-owning households were sampled, or where surveys were not administered.

## Appendix A

**Table A1:** A stepwise comparison of variance inflation factors (VIFs) for variables that may influence dog populations in Tanzania. Predictor variables with highest VIF values were removed, and the stepwise comparison repeated until all VIF values were below 5. NA = not applicable after the predictor was dropped.

Variables	VIF After Step			
	1	2	3	4
Human population in 2014	972.8	9.5	2.4	2.9
Number of households	9.5	NA	NA	NA
Proportion of livestock keeping households	8.8	7.8	5.4	NA
Number of people living in rural areas	948.9	2.9	NA	NA
Proportion of persons employed as livestock keepers	1.6	1.6	1.4	1.3
Setting (inland vs coastal)	1.9	1.9	1.8	1.5
Setting (mainland vs island)	9.2	5.3	4.7	2.1
Proportion of persons employed as peasant	9.4	8.8	5.4	4.7
Area	8.2	4.3	4.1	3.1

**Table A2:** Descriptive characteristics of the study districts. Number of households and employment status were obtained from the 2012 national census [16]. In household surveys, ~30 households/village were sampled in 6 (range 2–6) villages/district (ranged 93–180 households) per district ([13], Table 2). For school-based surveys, ~100 pupils were sampled per school in 6 (range 3–6) schools/district, whereby questionnaires were administered to 152–645 pupils per district. Dogs counted during surveys, mean dogs per household and vaccination coverage were compiled or calculated from household or school-based surveys. Human/dog ratios (HDRs) were calculated from transects and numbers of vaccinated dogs were compiled from mass dog vaccination campaigns, both conducted in 2014–2015. HHS = household survey. SBS = school-based survey. CI = confidence interval. NA = not applicable.

District	Setting	Number of Households (% Rural)	Employment Status in Percentage			HHS		SBS		HDR (CI) *	Vaccinated Dogs **	Estimated Dogs	
			Peasants	Livestock Keepers	Others	Dogs (Mean Dogs/HH)	Coverage (%)	Dogs (Mean Dogs/Pupil)	Coverage (%)			HHS	SBS
Chakechake	Coastal	17,551 (47)	51.1	0.5	48.4	7 (0.04)	0	3 (0.02)	100	59 (20, 177)	608	702	351
Ilala	Coastal	297,750 (9)	4.0	1.2	94.8	34 (0.26.)	50	NA	NA	NA	4218	77,415	NA
Kibaha Rural	Inland	16,892 (28)	47.2	5.8	47.0	30 (0.32)	23	199 (0.50)	59	13 (4, 40)	5226	5405	8446
Kibaha Urban	Inland	31,092 (25)	27.7	2.7	69.6	66 (0.44)	44	237 (0.51)	59	21 (7, 65)	3684	13,680	15,857
Kilombero	Inland	93,331 (37)	78.7	0.7	20.6	32 (0.22)	47	218 (0.40)	66	21 (7, 63)	14,208	20,533	37,332
Kilwa	Coastal	42,596 (48)	71.6	0.4	28.0	26 (0.16)	15	NA	NA	55 (18, 166)	2120	6815	NA
Kinondoni	Coastal	441,240 (8)	2.9	0.8	96.3	59 (0.32)	44	163 (0.35)	52	181(50, 648)	3696	141,197	154,434
Kisarawe	Inland	25,475 (48)	78.7	2.3	19.0	9 (0.05)	89	109 (0.39)	80	14 (4, 43)	2787	1274	9935
Lindi Rural	Coastal	52,821 (42)	87.2	0.1	12.7	15 (0.08)	60	60 (0.24)	55	83 (27, 257)	1148	4226	12,677
Lindi Urban	Coastal	22,344 (29)	55.8	0.3	43.9	17 (0.10)	41	70 (0.20)	50	68 (23, 205)	930	2234	4469
Liwale	Inland	21,084 (50)	76.2	0.1	23.7	19 (0.11)	16	NA	NA	43 (14, 132)	637	2319	NA
Masasi	Inland	67,872 (45)	82.2	0.1	17.7	27 (0.15)	30	32 (0.20)	69	42 (14, 127)	4558	10,181	13,574
Micheweni	Coastal	19,257 (52)	57.3	1.2	41.5	25 (0.14)	0	4 (0.03)	25	41 (14, 125)	569	2696	578
Mkoani	Coastal	18,067 (57)	26.0	0.6	73.4	9 (0.06)	100	8 (0.05)	100	75 (25, 224)	631	1084	903
Mkuranga	Coastal	51,101 (34)	68.5	0.5	31.0	4 (0.02)	100	58 (0.18)	62	52 (17, 157)	1811	1022	9198
Morogoro Rural	Inland	67,671 (46)	81.0	3.0	16.0	41 (0.24)	56	103 (0.25)	65	12 (4, 39)	6434	16,241	16,918
Morogoro Urban	Inland	76,039 (16)	25.2	0.7	74.1	49 (0.29)	76	225 (0.40)	67	35 (12, 108)	9968	22,051	30,416
Mtwara Rural	Inland	58,602 (37)	83.2	0.1	16.7	16 (0.11)	50	31 (0.09)	61	85 (27, 262)	860	6446	5274
Mtwara Urban	Coastal	27,968 (18)	27.7	0.4	71.9	14 (0.09)	64	69 (0.24)	35	84 (28, 252)	540	2517	6712
Nachingwea	Inland	48,145 (45)	85.7	0.1	14.2	37 (0.22)	32	84 (0.25)	8	41 (12, 125)	2823	10,592	12,036
Nanyumbu	Inland	40,746 (33)	88.4	0.1	11.5	1 (0.01)	100	28 (0.06)	18	41 (13, 123)	1281	407	2445
Newala	Inland	58,035 (49)	81.2	0.1	18.7	4 (0.02)	100	55 (0.09)	53	42 (14, 128)	1465	1161	5223
Ruangwa	Inland	37,326 (47)	83.7	0.1	16.2	37 (0.21)	35	24 (0.14)	50	42 (14, 126)	1090	7838	5226
Rufiji	Coastal	48,164 (31)	77.1	1.3	21.6	2 (0.01)	100	61 (0.14)	49	35 (11, 109)	1423	482	6743
Tandahimba	Inland	60,872 (44)	86.2	0.2	13.6	2 (0.03)	100	24 (0.14)	8	32 (11, 95)	762	1826	8522
Temeke	Coastal	344,391 (6)	4.6	0.8	94.6	8 (0.05)	88	NA	NA	147 (44, 496)	2521	17,220	NA
Ulanga	Inland	53,290 (42)	83.2	1.1	15.7	85 (0.48)	45	326 (0.58)	61	18 (6, 53)	9645	25,579	30,908
Wete	Coastal	20,151 (40)	47.2	0.8	52.0	56 (0.38)	57	7 (0.04)	100	52 (17, 155)	718	7657	806
Overall						731 (0.6)	56	2,198 (0.7)	56		86,321	410,800	398,983
